# Autonomic nervous alterations associated with daily level of fatigue

**DOI:** 10.1186/1744-9081-7-46

**Published:** 2011-10-27

**Authors:** Masaaki Tanaka, Kei Mizuno, Kouzi Yamaguti, Hirohiko Kuratsune, Akira Fujii, Hiromichi Baba, Kazuya Matsuda, Ayako Nishimae, Toshio Takesaka, Yasuyoshi Watanabe

**Affiliations:** 1 Department of Physiology, Osaka City University Graduate School of Medicine, 1-4-3 Asahimachi, Abeno-ku, Osaka 545-8585, Japan; 2 Molecular Probe Dynamics Laboratory, RIKEN Center for Molecular Imaging Science, 6-7-3 Minatojima-minamimachi, Chuo-ku, Hyogo 650-0047, Japan; 3 Department of Health Science, Faculty of Health Science for Welfare, Kansai University of Welfare Sciences, 3-11-1 Asahigaoka, Osaka 582-0026, Japan; 4 Health Chemical Co., Ltd., 179 Ikeda, Kume, Tokoname City, Aichi 479-0002, Japan; 5 Osaka Urban Industry Promotion Center, 1-4-5 Honmachi, Chuo-ku, Osaka City, Osaka 541-0053, Japan

**Keywords:** Advanced trail making test, 2-back Test, Parasympathetic nerve function, Selective attention, Sympathetic nerve function

## Abstract

**Background:**

Fatigue is a common symptom in both sick and healthy people. We examined autonomic nervous alterations associated with fatigue to clarify the mechanisms underlying fatigue.

**Methods:**

The study group consisted of 19 healthy participants who performed a 2-back test for 30 min as a fatigue-inducing mental task session. Before and after the session, they completed the advanced trail making test (ATMT) for 30 min for mental fatigue evaluation, subjective scales to measure fatigue sensation, and underwent electrocardiography to allow assessment of autonomic nerve activities.

**Results:**

After the fatigue-inducing task, the total error counts on the ATMT tended to increase (*P *= 0.076); the ATMT for total trial counts (*P *= 0.001), the subjective level of fatigue (*P *< 0.001), and the % low-frequency power (%LF) (*P *= 0.035) increased significantly; and the % high-frequency power (%HF) decreased compared with before the fatigue-inducing task although this did not reach the statistical significance (*P *= 0.170). Although LF measured in absolute units did not change significantly before and after the fatigue-inducing task (*P *= 0.771), and HF measured in absolute units decreased after the task (*P *= 0.020). The %LF and LF/HF ratio were positively associated with the daily level of fatigue evaluated using Chalder's fatigue scale. In addition, %HF was negatively associated with the fatigue score.

**Conclusions:**

Increased sympathetic activity and decreased parasympathetic activity may be characteristic features of both acute and daily levels of fatigue. Our findings provide new perspectives on the mechanisms underlying fatigue.

## Background

Many people experience fatigue after or during a prolonged period of activity [1]. Large community surveys have reported that up to half of the general adult population complains of fatigue [2,3]. In Japan, more than half of the general adult population complains of fatigue, and more than one third of the population suffers from chronic fatigue [4]. Acute fatigue is a normal phenomenon that disappears after a period of rest; in contrast, chronic fatigue is sometimes irreversible and the compensation mechanisms that are useful in reducing acute fatigue are not effective [5]. Therefore, it is important to clarify the mechanisms underlying fatigue, and in particular, long-term fatigue.

Fatigue-related alterations of autonomic nerve activities have been reported in patients with chronic fatigue syndrome (CFS) [6-11], multiple sclerosis [12-14], and primary biliary cirrhosis [9,15]. These reports suggest that changes in autonomic nerve activity are related to the mechanisms underlying fatigue. However, this relationship has been demonstrated only in patients with specific diseases and not in healthy subjects.

Recently, we demonstrated that decreased parasympathetic activity and increased sympathetic activity were induced in healthy volunteers following a 30-min fatigue-inducing mental task session [16]. As chronic or daily levels of fatigue can be evaluated using a paper-and-pencil questionnaire [17], the relationships between daily levels of fatigue and alterations of autonomic nerve activities may be identified. In addition, we can evaluate acute and daily levels of fatigue simultaneously in the same participants by using previously performed fatigue-inducing and fatigue-evaluating experiments [16]. The aim of the present study was to determine alterations in autonomic nerve activities associated with daily levels of fatigue as well as acute fatigue.

## Methods

### Participants

Nineteen healthy volunteers (mean age, 43.6 ± 10.1 years; 15 women and 4 men) were enrolled. None of the participants had a history of medical illness. Participants with a history of health problems, taking chronic medication or supplemental vitamins, and those who weighed < 40 kg [18-22] were excluded. Good health was assessed by physical examination, blood pressure, and heart rate. The protocol was approved by the Ethics Committee of Osaka City University, and all participants provided written informed consent.

### Experimental design

The day before the experiment, participants finished dinner by 9:00 pm and then fasted overnight. The following morning, they had breakfast before the visit. At 10:00 a.m., after the visit, they started the experiment. Before the start of the experiment, a paper-and-pencil questionnaire was distributed to participants to evaluate their daily level of fatigue. As a fatigue-inducing mental task session, participants performed 2-back test [23] trials for 30 min [24], and as a fatigue-evaluating mental task, they performed the advanced trail making test (ATMT; [25]) for 30 min [24] before and after the fatigue-inducing task session. Just before and after the fatigue-inducing session, they recorded their subjective sensation of fatigue on a visual analogue scale (VAS) from 0 (no fatigue) to 100 (complete exhaustion) [26] and underwent electrocardiography (ECG) with their eyes closed for 1 min while sitting on a chair. VAS and ECG recordings were performed before the ATMT trials. This study was conducted in a quiet, temperature- and humidity-controlled environment. For 1 day before the visit, participants refrained from intense mental and physical activities and caffeinated beverages, consumed a normal diet, and maintained normal sleeping hours.

### Questionnaire

A paper-and-pencil questionnaire was distributed to participants. The severity of daily level of fatigue was measured using Chalder's fatigue scale (Chalder et al. 1993), which has been previously used in Japanese participants [27]. The reliability and validity of the Japanese version of Chalder's fatigue scale to evaluate the severity of daily fatigue have been previously confirmed [27]. The fatigue scale consists of 11 questions using a 4-point (0-3) Likert scale that allows the following responses: 0 = less than usual; 1 = no more than usual; 2 = more than usual; 3 = much more than usual during the past several weeks. The total score for the 11-item fatigue scale ranges from 0 to 33, with higher scores indicating a greater degree of daily fatigue.

### Fatigue-inducing mental task

As a fatigue-inducing mental task, participants performed the 2-back test for 30 min [24]. During this task, one of four letters was presented on a display of a personal computer every 3 sec, and they had to judge whether the target letter presented at the center of the screen was the same as the one that had appeared 2 presentations before. If it was, they were to press the right mouse button with their right middle finger; if it was not, they were to press the left mouse button with their right index finger. They were instructed to perform the task trials as quickly and as correctly as possible. The results of each 2-back trial, that is, a correct response or error, were continuously presented on the display of the personal computer.

### Fatigue-evaluating mental task

As a fatigue-evaluating mental task, participants performed the ATMT for 30 min [24]. In this test, circles numbered from 1 to 25 were randomly placed on the display of a personal computer, and participants were required to use a computer mouse to touch these circles in sequence, starting with number 1. Tasks A, B, and C all ended when they touched the 25th target. They continued directly with the next Tasks B, C, and A, in that order, on and on for 30 min. The number of hits counted and the time were counted. In task A of the ATMT, when they touched a target circle, it remained in the same position, but the color changed from black to yellow. The positions of the other circles remained the same. In task B of the ATMT, when they touched the first target circle, it disappeared, and circle number 26 appeared in a different position on the screen. The positions of the other circles remained the same. For example, touching circles 2, 3, and 4 resulted in their disappearance and the addition of circles 27, 28, and 29 on the screen, so that there were always 25 circles on the screen. In task C of the ATMT, when they touched the first target circle, it disappeared and circle number 26 appeared in a different position on the screen and the position of all other circles changed at random. As in task B, there were always 25 circles on the screen. Participants performed tasks A, B, and C consecutively. They were instructed to perform all task trials as quickly and as correctly as possible.

### Electrocardiographic analyses

ECG was recorded using active tracer AC301 (Global Medical Solution Inc., Tokyo, Japan), and the ECG was analyzed using MemCalc for Windows (Global Medical Solution Inc.). Data were analyzed offline after analogue-to-digital conversion at 250 Hz. R-R wave. Irregularities in the ECG recordings were excluded from the analyses. Variability was measured as an indicator of autonomic nerve activity. For frequency domain analyses of the R-R wave intervals, the percent of low-frequency power (LF) was calculated as the power within the frequency range of 0.04 to 0.15 Hz, and the percent of high-frequency power (HF) was calculated as that within the frequency range of 0.15 to 0.4 Hz. LF and HF were measured in absolute and normalized units; normalization was performed by dividing the absolute power by the total variance and then multiplying by 100. The %HF is vagally mediated [28-30], but the %LF originates from a variety of sympathetic and vagal mechanisms [28,31]. The LF/HF ratio represents the sympathetic to parasympathetic balance [32].

### Statistical analysis

Values are shown as mean ± SD unless otherwise noted. Paired t-tests were used to evaluate the differences before and after the mental fatigue-inducing task as for the ATMT performances and VAS scores and Wilcoxon's signed rank tests as for the indices of the heart rate variability. Pearson's correlation analyses were conducted to evaluate relationships between two variables. All *P *values were 2-tailed, and *P *values less than 0.05 were considered statistically significant. Statistical analyses were performed using SPSS 17.0 software package (SPSS Inc., Chicago, IL).

## Results

Task performances, subjective level of fatigue, and ECG variables before and after the fatigue-inducing mental task are shown in Table 1. After the fatigue-inducing task, the total error counts of the ATMT during the fatigue-evaluating mental task tended to increase compared with before the fatigue-inducing task, although differences did not reach statistical significance (*P *= 0.076). In addition, after the fatigue-inducing task, the total trial counts (sum of the counts to touch the circles in sequence) of the ATMT (*P *= 0.001) indicating that they became faster after the fatigue-inducing task, subjective level of fatigue (*P *< 0.001), and %LF (*P *= 0.035) increased significantly, whereas %HF decreased although this did not reach statistical significance (*P *= 0.170). Although LF measured in absolute units did not change significantly before and after the fatigue-inducing task (*P *= 0.771), HF measured in absolute units decreased after the task (*P *= 0.020). The LF/HF ratio did not change significantly before and after the fatigue-inducing task (*P *= 0.805).

**Table 1:** Effect of acute mental fatigue on various parameters

	Before	After	*P *value
ATMT for total error counts	16 ± 19	20 ± 26	0.076
ATMT for total trial counts	305 ± 53	325 ± 45	0.001
VAS for fatigue	21 ± 13	47 ± 20	< 0.001
ECG variables			
%LF	38 ± 16	48 ± 18	0.035
%HF	40 ± 20	33 ± 17	0.170
LF/HF	3.4 ± 7.1	3.0 ± 3.8	0.396

Data are mean ± SD.

ATMT, advanced trail making test; VAS, visual analogue scale; ECG, electrocardiography; %LF, % low-frequency power; %HF, % high-frequency power.

**Figure 1:**
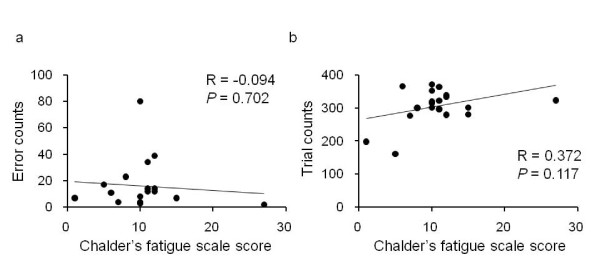
**Relationships between Chalder's fatigue scale score and task performances**. The task performances were assessed using total error counts (a) and total trial counts (b) of the fatigue-evaluating mental task. Linear regression lines, Pearson's correlation coefficients (R), and *P *values are shown.

Relationships between Chalder's fatigue scale score and task performances on the ATMT before the fatigue-inducing mental task are shown in Figure 1. The total error and trial counts were not associated with the Chalder's fatigue scale score.

**Figure 2:**
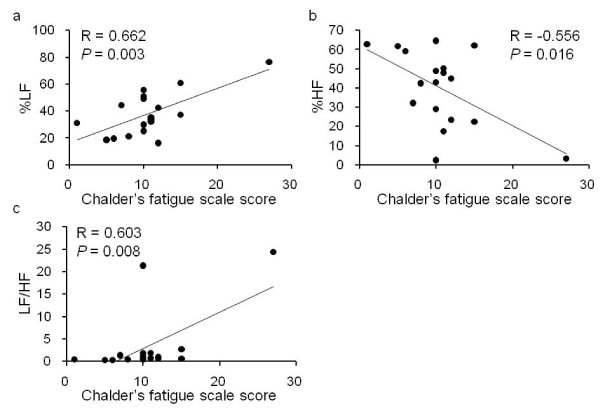
**Relationships between Chalder's fatigue scale score and autonomic nerve activities**. Autonomic nerve activities were evaluated using the % low-frequency power (%LF; a), % high-frequency power (%HF; b), and LF/HF ratio (c) obtained on R-R wave interval analyses using electrocardiography. Linear regression lines, Pearson's correlation coefficients (R), and *P *values are shown.

Relationships between Chalder's fatigue scale score and ECG variables before the fatigue-inducing mental task are shown in Figure 2. %LF and LF/HF ratio were positively associated with the Chalder's fatigue scale score, and %HF was negatively associated with the fatigue score.

## Discussion

The present study showed that, after an acute fatigue-inducing mental task, subjective levels of fatigue, %LF, and total error counts on the ATMT (tendency) increased and %HF decreased (although this did not reach statistical signigicance) compared with before the fatigue-inducing task. In addition, %LF and LF/HF ratio were positively, and %HF was negatively associated with the Chalder's fatigue scale score.

These findings are consistent with the results of our previous report [27], in which decreased parasympathetic and increased sympathetic activities were caused after a 30-min fatigue-inducing mental task. The brain network, including the prefrontal cortex (PFC) and anterior cingulate cortex (ACC), has been shown to play an important role in the regulation of autonomic nervous activities [33]. Decreased parasympathetic activity and increased sympathetic activity are interpreted as a state of autonomic hypervigilance [34,35], and sympathoexcitatory subcortical circuits are normally under the inhibitory control of the PFC [34-36]. In addition, the ACC is related to the regulation of parasympathetic activity [37,38]. Because impaired selective attention assessed by increased error counts of the ATMT [24] was observed after the fatigue-inducing task, and the selective attention process activates the PFC and ACC [39-42], acute mental load might introduce temporary dysfunctions in the PFC and ACC to cause decreased parasympathetic and increased sympathetic activities.

Decreased parasympathetic nerve activity and increased sympathetic activity have also been observed in patients with CFS [10,11,43]. Because bilateral reduction of grey-matter volume in the PFC [44] and decreased cerebral blood flow [45] and reduction of serotonin transporters [46] in the ACC were reported in the patients with CFS, decreased parasympathetic and increased sympathetic activities may be induced by the chronic anatomical and/or functional alterations in the PFC and ACC in these patients. Hence, chronic fatigue is characterized as decreased parasympathetic and increased sympathetic activities, and the pathophysiological background may be explained by chronic alterations in the PFC and ACC.

## Limitations

The present study has several limitations. First, the study included a small number of participants. In addition, we did not obtain the information as for such as smoking habit or lifestyles, and a great majority of women was included maybe because we recruited the participants via advertisement. Studies involving a larger number of participants and more detailed information regarding the participants are needed to allow for generalization of these results. Second, conclusions about cause-and-effect relationships cannot be made due to the cross-sectional nature of the data. Third, heart variability indices are measures of autonomic modulation of the sinus node-not autonomic tone. Heart rate variability indices must be interpreted in light of the heart rate itself. Finally, the daily level of fatigue was evaluated using self-reports, and as such, was subjective. An objective biomarker for daily level of fatigue has been developed [47]. The number of saliva human herpesvirus (HHV)-6 DNA copies was decreased after holidays for approximately 1 week [47]. The reliability and validity of the results of this study should be confirmed using this biomarker.

## Conclusions

The present results provide evidence that increased sympathetic activity and decreased parasympathetic activity are associated with both the acute and daily level of fatigue. Because increased sympathetic activity and decreased parasympathetic activity have been reported in patients with CFS [10,11,43], these alterations of the autonomic nerve activities may be common characteristics of fatigue. Based on these findings, transitional mechanisms from acute fatigue to chronic fatigue and chronic fatigue to chronic fatigue-associated disease might be clarified. Our findings provide new perspectives on the mechanisms underlying fatigue.

## List of abbreviations

ACC: Anterior cingulate cortex; ATMT: Advanced trail making test; CFS: chronic fatigue syndrome; ECG: Electrocardiography; HF: High-frequency power; HHV: Human herpesvirus; LF: Low-frequency power; PFC: Prefrontal cortex; VAS: Visual analogue scale.

## Competing interests

The authors declare that they have no competing interests.

## Authors' contributions

MT took part in planning and designing the experiment and cognitive tests, collected the data, performed the data analyses and drafted the manuscript. KM, KY, and HK contributed to the design, planning of experiment and cognitive tests, and helped performing the data analyses. AF, HB, AN, and TT contributed to the design, planning of experiment, and collected the data. YW took part in the planning and designing the experiment and cognitive tests and helped drafting the manuscript. All authors read and approved the final manuscript.

## References

[B1] Boksem MA Tops M Mental fatigue: costs and benefits Brain Res Rev 2008 59 1 125 139 10.1016/j.brainresrev.2008.07.001 18652844

[B2] Chen MK The epidemiology of self-perceived fatigue among adults P rev Med 1986 15 1 74 81 10.1016/0091-7435(86)90037-x 3714661

[B3] Pawlikowska T Chalder T Hirsch SR Wallace P Wright DJ Wessely SC Population based study of fatigue and psychological distress BMJ 1994 308 6931 763 766 10.1136/bmj.308.6931.763 7908238 PMC2539651

[B4] Watanabe Y Watanabe Y, Evengård B, Natelson BH, Jason LA, Kuratsune H Preface and mini-review: fatigue science for human health Fatigue Science for Human Health 2008 New York: Springer 5 11

[B5] Beurskens AJ Bültmann U Kant I Vercoulen JH Bleijenberg G Swaen GM Fatigue among working people: validity of a questionnaire measure Occup Environ Med 2000 57 5 353 357 10.1136/oem.57.5.353 10769302 PMC1739950

[B6] Stewart JM Autonomic nervous system dysfunction in adolescents with postural orthostatic tachycardia syndrome and chronic fatigue syndrome is characterized by attenuated vagal baroreflex and potentiated sympathetic vasomotion Pediatr Res 2000 48 2 218 226 10.1203/00006450-200008000-00016 10926298

[B7] Freeman R The chronic fatigue syndrome is a disease of the autonomic nervous system. Sometimes Clin Auton Res 2002 12 4 231 233 10.1007/s10286-002-0058-2 12357274

[B8] Winkler AS Blair D Marsden JT Peters TJ Wessely S Cleare AJ Autonomic function and serum erythropoietin levels in chronic fatigue syndrome J Psychosom Res 2004 56 2 179 183 10.1016/S0022-3999(03)00543-9 15016575

[B9] Newton JL Okonkwo O Sutcliffe K Seth A Shin J Jones DE Symptoms of autonomic dysfunction in chronic fatigue syndrome QJM 2007 100 8 519 526 10.1093/qjmed/hcm057 17617647

[B10] Wyller VB Saul JP Amlie JP Thaulow E Sympathetic predominance of cardiovascular regulation during mild orthostatic stress in adolescents with chronic fatigue Clin Physiol Funct Imaging 2007 27 4 231 238 10.1111/j.1475-097X.2007.00743.x 17564672

[B11] Burton AR Rahman K Kadota Y Lloyd A Vollmer-Conna U Reduced heart rate variability predicts poor sleep quality in a case-control study of chronic fatigue syndrome Exp Brain Res 2010 204 1 71 78 10.1007/s00221-010-2296-1 20502886

[B12] Keselbrener L Akselrod S Ahiron A Eldar M Barak Y Rotstein Z Is fatigue in patients with multiple sclerosis related to autonomic dysfunction? Clin Auton Res 2000 10 4 169 175 10.1007/BF02291352 11029013

[B13] Merkelbach S Dillmann U Kölmel C Holz I Muller M Cardiovascular autonomic dysregulation and fatigue in multiple sclerosis Mult Scler 2001 7 5 320 326 11724448 10.1177/135245850100700508

[B14] Flachenecker P Rufer A Bihler I Hippel C Reiners K Toyka KV Kesselring J Fatigue in MS is related to sympathetic vasomotor dysfunction Neurology 2003 61 6 851 853 14504339 10.1212/01.wnl.0000080365.95436.b8

[B15] Newton JL Davidson A Kerr S Bhala N Pairman J Burt J Jones DE Autonomic dysfunction in primary biliary cirrhosis correlates with fatigue severity Eur J Gastroenterol Hepatol 2007 19 2 125 132 10.1097/01.meg.0000252629.96043.67 17272997

[B16] Tanaka M Mizuno K Tajima S Sasabe T Watanabe Y Central nervous system fatigue alters autonomic nerve activity Life Sci 2009 84 7-8 235 239 10.1016/j.lfs.2008.12.004 19100749

[B17] Chalder T Berelowitz G Pawlikowska T Watts L Wessely S Wright D Wallace EP Development of a fatigue scale J Psychosom Res 1993 37 2 147 153 10.1016/0022-3999(93)90081-P 8463991

[B18] Ataka S Tanaka M Nozaki S Mizuma H Mizuno K Tahara T Sugino T Shirai T Kajimoto Y Kuratsune H Kajimoto O Watanabe Y Effects of Applephenon and ascorbic acid on physical fatigue Nutrition 2007 23 5 419 423 10.1016/j.nut.2007.03.002 17483009

[B19] Ataka S Tanaka M Nozaki S Mizuma H Mizuno K Tahara T Sugino T Shirai T Kajimoto Y Kuratsune H Kajimoto O Watanabe Y Effects of oral administration of caffeine and D-ribose on mental fatigue Nutrition 2008 24 3 233 238 10.1016/j.nut.2007.12.002 18178380

[B20] Mizuno K Tanaka M Nozaki S Mizuma H Ataka S Tahara T Sugino T Shirai T Kajimoto Y Kuratsune H Kajimoto O Watanabe Y Antifatigue effects of coenzyme Q10 during physical fatigue Nutrition 2008 24 4 293 299 10.1016/j.nut.2007.12.007 18272335

[B21] Mizuma H Tanaka M Nozaki S Mizuno K Tahara T Ataka S Sugino T Shirai T Kajimoto Y Kuratsune H Kajimoto O Watanabe Y Daily oral administration of crocetin attenuates physical fatigue in human subjects Nutr Res 2009 29 3 145 150 10.1016/j.nutres.2009.02.003 19358927

[B22] Nozaki S Tanaka M Mizuno K Ataka S Mizuma H Tahara T Sugino T Shirai T Eguchi A Okuyama K Yoshida K Kajimoto Y Kuratsune H Kajimoto O Watanabe Y Mental and physical fatigue-related biochemical alterations Nutrition 2009 25 1 51 57 10.1016/j.nut.2008.07.010 18834718

[B23] Braver TS Cohen JD Nystrom LE Jonides J Smith EE Noll DC A parametric study of prefrontal cortex involvement in human working memory Neuroimage 1997 5 1 49 62 10.1006/nimg.1996.0247 9038284

[B24] Mizuno K Watanabe Y Watanabe Y, Evengård B, Natelson BH, Jason LA, Kuratsune H Utility of an advanced trail making test as a neuropsychological tool for an objective evaluation of work efficiency during mental fatigue Fatigue Science for Human Health 2008 New York: Springer 47 54

[B25] Kajimoto O Watanabe Y, Evengård B, Natelson BH, Jason LA, Kuratsune H Development of a method of evaluation of fatigue and its economic impacts Fatigue Science for Human Health 2008 New York: Springer 33 46

[B26] Lee KA Hicks G Nino-Murcia G Validity and reliability of a scale to assess fatigue Psychiatry Res 1991 36 3 291 298 10.1016/0165-1781(91)90027-M 2062970

[B27] Tanaka M Fukuda S Mizuno K Kuratsune H Watanabe Y Stress and coping styles are associated with severe fatigue in medical students Behav Med 2009 35 3 87 92 19812026 10.1080/08964280903231979

[B28] Akselrod S Gordon D Ubel FA Shannon DC Berger AC Cohen RJ Power spectrum analysis of heart rate fluctuation: a quantitative probe of beat-to-beat cardiovascular control Science 1981 213 4504 220 222 10.1126/science.6166045 6166045

[B29] Pomeranz B Macaulay RJ Caudill MA Kutz I Adam D Gordon D Kilborn KM Barger AC Shannon DC Cohen RJ Assessment of autonomic function in humans by heart rate spectral analysis Am J Physiol 1985 248 1 Pt 2 151 153 10.1152/ajpheart.1985.248.1.H151 3970172

[B30] Malliani A Pagani M Lombardi F Cerutti S Cardiovascular neural regulation explored in the frequency domain Circulation 1991 84 2 482 492 1860193 10.1161/01.cir.84.2.482

[B31] Appel ML Berger RD Saul JP Smith JM Cohen RJ Beat to beat variability in cardiovascular variables: noise or music? J Am Coll Cardiol 1989 14 5 1139 1148 10.1016/0735-1097(89)90408-7 2681319

[B32] Pagani M Montano N Porta A Malliani A Abboud FM Birkett C Somers VK Relationship between spectral components of cardiovascular variabilities and direct measures of muscle sympathetic nerve activity in humans Circulation 1997 95 6 1441 1448 9118511 10.1161/01.cir.95.6.1441

[B33] Tang YY Ma Y Fan Y Feng H Wang J Feng S Lu Q Hu B Lin Y Li J Zhang Y Wang Y Zhou L Fan M Central and autonomic nervous system interaction is altered by short-term meditation Proc Natl Acad Sci USA 2009 106 22 8865 8870 10.1073/pnas.0904031106 19451642 PMC2690030

[B34] Thayer JF On the importance of inhibition: central and peripheral manifestations of nonlinear inhibitory processes in neural systems Dose Response 2006 4 1 2 21 10.2203/dose-response.004.01.002.Thayer 18648636 PMC2477656

[B35] Thayer JF Sternberg E Beyond heart rate variability: vagal regulation of allostatic systems Ann N Y Acad Sci 2006 1088 361 372 10.1196/annals.1366.014 17192580

[B36] Amat J Baratta MV Paul E Bland ST Watkins LR Maier SF Medial prefrontal cortex determines how stressor controllability affects behavior and dorsal raphe nucleus Nat Neurosci 2005 8 3 365 371 10.1038/nn1399 15696163 PMC13675003

[B37] Kubota Y Sato W Toichi M Murai T Okada T Hayashi A Sengoku A Frontal midline theta rhythm is correlated with cardiac autonomic activities during the performance of an attention demanding meditation procedure Brain Res Cogn Brain Res 2001 11 2 281 287 10.1016/S0926-6410(00)00086-0 11275489

[B38] Takahashi M Arito H Effects of single and repeated cognitive tasks on autonomic balance as observed by an analysis of R-R intervals Eur J Appl Physiol Occup Physiol 1996 72 4 316 322 10.1007/BF00599691 8851900

[B39] Danckert J Maruff P Ymer C Kinsella G Yucel M de Graaff S Currie J Goal-directed selective attention and response competition monitoring: evidence from unilateral parietal and anterior cingulate lesions Neuropsychology 2000 14 1 16 28 10674795 10.1037//0894-4105.14.1.16

[B40] Weissman DH Giesbrecht B Song AW Mangun GR Woldorff MG Conflict monitoring in the human anterior cingulate cortex during selective attention to global and local object features Neuroimage 2003 19 4 1361 1368 10.1016/S1053-8119(03)00167-8 12948694

[B41] Schreppel TJ Pauli P Ellgring H Fallgatter AJ Herrmann MJ The impact of prefrontal cortex for selective attention in a visual working memory task Int J Neurosci 2008 118 12 1673 88 10.1080/00207450601067356 18937114

[B42] Morishima Y Akaishi R Yamada Y Okuda J Toma K Sakai K Task-specific signal transmission from prefrontal cortex in visual selective attention Nat Neurosci 2009 12 1 85 91 10.1038/nn.2237 19098905

[B43] Yamaguti K Sasabe T Kuratsune H Nishizawa Y Watanabe Y The Journal of Therapy 2008 90 537 547 (In Japanese)

[B44] Okada T Tanaka M Kuratsune H Watanabe Y Sadato N Mechanisms underlying fatigue: a voxel-based morphometric study of chronic fatigue syndrome BMC Neurol 2004 4 1 14 10.1186/1471-2377-4-14 15461817 PMC524491

[B45] Kuratsune H Yamaguti K Lindh G Evengård B Hagberg G Matsumura K Iwase M Onoe H Takahashi M Machii T Kanakura Y Kitani T Långström B Watanabe Y Brain regions involved in fatigue sensation: reduced acetylcarnitine uptake into the brain Neuroimage 2002 17 3 1256 1265 10.1006/nimg.2002.1260 12414265

[B46] Yamamoto S Ouchi Y Onoe H Yoshikawa E Tsukada H Takahashi H Iwase M Yamaguti K Kuratsune H Watanabe Y Reduction of serotonin transporters of patients with chronic fatigue syndrome Neuroreport 2004 15 17 2571 2574 10.1097/00001756-200412030-00002 15570154

[B47] Kondo K Human herpesvirus latency and fatigue Uirusu 2005 55 9 17 (In Japanese) 10.2222/jsv.55.9 16308525

